# Risk of Cancer Following the Use of N-Nitrosodimethylamine (NDMA) Contaminated Ranitidine Products: A Nationwide Cohort Study in South Korea

**DOI:** 10.3390/jcm10010153

**Published:** 2021-01-05

**Authors:** Hong Jin Yoon, Jie-Hyun Kim, Gi Hyeon Seo, Hyojin Park

**Affiliations:** 1 Department of Internal Medicine, Soonchunhyang University College of Medicine, Cheonan-si, Chungcheongnam-do 330-921, Korea; 104560@schmc.ac.kr; 2 Department of Internal Medicine, Gangnam Severance Hospital, Yonsei University College of Medicine, 211 Eonjuro, Gangnam-gu, Seoul 135-720, Korea; hjpark21@yuhs.ac; 3 Health Insurance Review and Assessment Service, 60, Hyeoksin-ro, Wonju-si, Gangwon-do 26465, Korea

**Keywords:** ranitidine, NDMA, cancer risk, claims data, famotidine

## Abstract

N-nitrosodimethylamine (NDMA), a known carcinogenic agent, was recently detected in some products of ranitidine. Several studies have investigated the detectability of NDMA, in drugs and their risks. However, only a few epidemiological studies have evaluated cancer risk from the use of such individual drugs. This study investigates the risk of cancer in ranitidine users. We conducted an observational population-based cohort study using the Health Insurance Review and Assessment databases, which contain information about the use of medicines in South Korea. The primary study cohort consisted of ranitidine users (n = 88,416). For controls, we enrolled users of famotidine, another H_2_-receptor antagonist in which no NDMA has been detected. A 4:1 matched cohort was constructed to compare cancer outcomes of the two groups. Our matched cohort comprised of 40,488 ranitidine users and 10,122 famotidine users. There was no statistical difference in the overall cancer risk between the ranitidine and famotidine groups (7.45% vs. 7.56%, HR 0.99, 95% CI 0.91–1.07, *p* = 0.716). Additionally, no significant differences were observed in the analysis of 11 single cancer outcomes. We found no evidence that exposure to NDMA through ranitidine increases the risk of cancer.

## 1. Introduction

N-nitrosodimethylamine (NDMA) is a known environmental contaminant found in water, foods (such as dairy products, vegetables, grilled meats), and several industrial processes [1,2]. There has been no study investigating the carcinogenic effects in humans following oral exposure of NDMA. However, many animal studies have demonstrated the carcinogenic effects of NDMA [3]. NDMA has been classified by the International Agency for Research on Cancer (IRAC) as a probable human carcinogen (group 2A) [4].

Ranitidine is a H_2_-receptor antagonist (H2RA) that is used widely to reduce the production of gastric acid in patients with gastroesophageal reflux disease (GERD) and peptic ulcer disease (PUD) [5]. In September 2019, the U.S. Food and Drug Administration (FDA) and the European Medicine Agency announced that NDMA was detected in Zantac^®^, one of the trade names of ranitidine. FDA had set the acceptable daily intake limit for NDMA at 0.096 micrograms or 0.32 ppm for ranitidine. However, testing of ranitidine products showed that NDMA levels were as much as nine times greater than the FDA’s recommended limit. Therefore, many manufacturers and retailers have voluntarily recalled ranitidine worldwide. Not long after, NDMA was detected in another antihistamine, nizatidine [6].

Previously, NDMA was identified in medicines containing valsartan in June 2018, and FDA recalled many batches of valsartan products from the global market. NDMA is believed to have been introduced into valsartan products due to the manufacturing process of the active pharmaceutical ingredient.

Several studies have detected N-nitrosamine precursors, including NDMA, in drugs, and investigated their risks [7,8,9,10,11]. However, only a few epidemiological studies have evaluated cancer risk from the use of such drugs [12]. Pharmacoepidemiologic studies are needed for clinically relevant causality assessment. For this purpose, a cohort study that identifies the patients’ individual medication and monitors the side effects through long-term follow-up is appropriate.

Therefore, the present study aimed to investigate the association between the use of ranitidine containing NMDA and the risk of cancer.

## 2. Methods

### 2.1. Data Source

We conducted an observational population-based cohort study using the Health Insurance Review and Assessment (HIRA) database in South Korea. HIRA contains health insurance claims data, also known as National Health Insurance Service (NHIS) data, because it is generated during the claims process for health care services in South Korea. The NHIS is a non-profit organization with a single insurer responsible for operating the health insurance program. Approximately 52 million residents in Korea are obliged to register with the NHIS. Insurance benefits of the coverage are extensive and include in-patient and out-patient care, prescription drugs, rehabilitation, and health promotion. Therefore, almost all medical practice is carried out with NHIS insurance, and the claims data for medical reimbursement will be requested to NHIS by the health care provider. As a result, all relevant data are accumulated in the NHIS database [13]. International Classification of Disease, Tenth Revision (ICD-10) codes are used for identifying the diagnosis. For patients with cancer, a special diagnostic code (C-code) is assigned in addition to the ICD-10 code. We investigated liver, lung, kidney, biliary tract, and prostate cancer, which has been shown to be associated with NDMA exposure in previous animal studies [14,15]. A total of 11 cancer outcomes were obtained from NHIS database, including those with high incidence. The cancer information collected is as follows: Primary malignant neoplasm of the liver (defined as ICD-10 codes C22), malignant neoplasm of the cecum, appendix, any site of the colon, and rectum (defined as ICD-10 codes C18–20), malignant neoplasm of the stomach (defined as ICD-10 codes C16), malignant neoplasm of the bronchus and lung (defined as ICD-10 codes C34), malignant neoplasm of the kidney, except renal pelvis (defined as ICD-10 codes C64), malignant neoplasm of any site in the bladder (defined as ICD-10 codes C67), malignant neoplasm of the uterus, myometrium, and cervix (defined as ICD-10 codes C53-54), malignant neoplasm of the breast (defined as ICD-10 codes C50), malignant neoplasm of the thyroid gland (defined as ICD-10 codes C73), malignant neoplasm of the gall bladder and biliary tract (defined as ICD-10 codes C23-24), and malignant neoplasm of the prostate (defined as ICD-10 codes C61). The C-code patients are charged a minimum amount of co-payments (5% of total costs) [16]. This study was conducted using NHIS data with approval from the Institutional Review Board of Gangnam Severance Hospital (no.3-2019-0318). 

### 2.2. Study Cohort

The study cohort consisted of patients prescribed ranitidine between January 2009 and December 2011. Patients who were prescribed famotidine during the same period were set as a comparison control group. Famotidine is an H2RA similar to ranitidine, but it does not contain NMDA as known from FDA tests. Individual periods of H2RA exposure were determined according to the intended duration of each prescription recorded in the database. We enrolled patients who were using ranitidine or famotidine for more than one year between January 2009 and December 2011. Ranitidine users of a cumulative dose exceeding 10,800 mg and famotidine users of a cumulative dose exceeding 14,400 mg were selected. To compare the equivalent doses between the two drugs, the defined daily dose (DDD) was calculated and compared. The DDD is the assumed average maintenance dose per day for a drug used for its main indication in adults [17].

Between January 2009 and December 2011, we identified 554,987 individuals prescribed ranitidine and 147,830 individuals prescribed famotidine. Of the total 702,817 patients, we excluded those who met any of the following criteria: (1) Users of ranitidine or famotidine between 2007–2008, (2) patients with an overlapping use of two drugs between 2009–2011, (3) diagnosed with cancer between 2007–2011, (4) aged under 30 or over 80, and (5) died before December 2011. 

After exclusion, a cohort of 88,416 ranitidine users and 10,129 famotidine users was enrolled, of which 42,488 ranitidine users and 10,122 famotidine users were selected for the study, in a ratio of 4:1. These groups were matched by sex, age, diabetes mellitus (DM), and cumulative exposure.

We excluded participants aged under 30 years as cancer occurrence is rare, and the types of cancer are often different in children and younger adults, from those that develop in older adults. In this study, we obtained cancer outcomes between January 2012 and December 2018 from the HIRA database. Cancer patients were identified by registered cancer diagnostic codes within a follow-up period. We investigated the occurrence of cancer in the lung, liver, kidney, biliary tract, and testis, which are organs with confirmed carcinogenicity of NDMA in animal models. In addition, the occurrence of cancer in the stomach, colorectal, thyroid gland, breast, uterus, and bladder, which also have a high incidence, was determined.

Within the study cohort, we used the unique drug ID recorded on the NHIS to verify exposure to ranitidine products in each participant and to identify the corresponding manufacturer. We further stratified the extent of NDMA exposure by the cumulative use of ranitidine and famotidine from the prescriptions (12–18 months, 18–24 months, 24–30 months, over 30 months).

### 2.3. Statistical Analysis

Continuous variables were analyzed using the Student’s *t*-test. Chi-squared test was used to detect the proportional differences between ranitidine and famotidine groups. A Cox proportional hazards regression model was used to assess the association between ranitidine or famotidine exposure and risk of cancer. For regression analyses, a hazard ratio (HR) with a 95% confidence interval (CI) was used. *p* < 0.05 was considered to indicate statistical significance. All statistical analyses were performed using R Statistical Software (version 3.5.0; R Foundation for Statistical Computing, Vienna, Austria).

### 2.4. Patient and Public Involvement 

No patients were involved in setting the research question, outcome measures, the study design, or the conduct of the study. Patients were not invited to contribute to the writing or editing of this document for readability or accuracy.

## 3. Results

**Figure 1:**
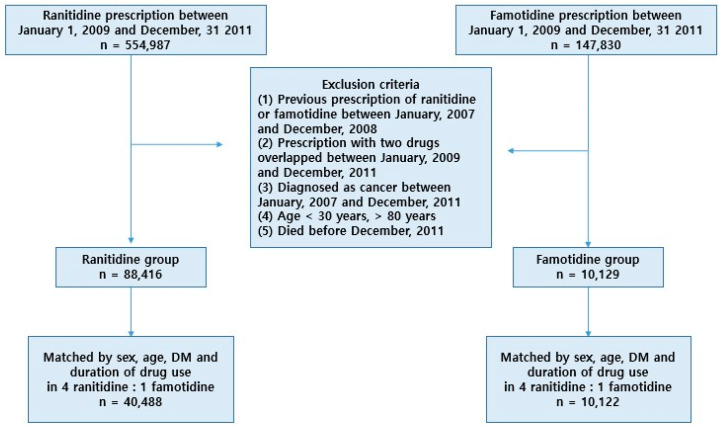
Flowchart of 4:1 matched cohort assembly of ranitidine users (*n =* 40,488) and famotidine users (*n* = 10,122).

There were 88,416 and 10,129 subjects in the ranitidine and famotidine groups, respectively (Figure 1). The baseline characteristics of the two groups are listed in Table 1. During the study period, the average cumulative dosage of ranitidine was 220,394 mg, and the average DDD was 683 days. In the famotidine group, the average cumulative dosage was 29,578 mg, and DDD was 739 days. DM was also found to be present in 36.3% of ranitidine users and 34.6% of famotidine users. In terms of cumulative duration, 12–17 months were the most frequent in both groups (45.9% vs. 39.7%). However, 19.6% of the ranitidine group and 25.8% of the famotidine group were used for more than 30 months. 

We examined the risk of cancer outcomes for a 4:1 matched cohort of users of ranitidine (*n* = 40,488) and famotidine (*n* = 10,122). The flowchart for the cohort design is provided in Figure 1. Subjects from the ranitidine group that were statistically matched to those from the famotidine group were selected considering the following factors: Age, sex distribution, cumulative duration, DM, and DM period. DM and DM period were included as matching factors because DM is a known risk factor for most cancers. Table 2 shows the baseline characteristics of the 4:1 well-balanced, matched cohort. The incidence for overall cancer was 3018 (7.45%) in the ranitidine group and 765 (7.56%) in the famotidine group. There was no statistical difference in overall cancer risk between the ranitidine and famotidine groups (HR 0.99, 95% CI 0.91 to 1.07, *p* = 0.716). Furthermore, no significant differences were observed in 11 single cancer outcomes between the two groups (Table 3). 

**Figure 2:**
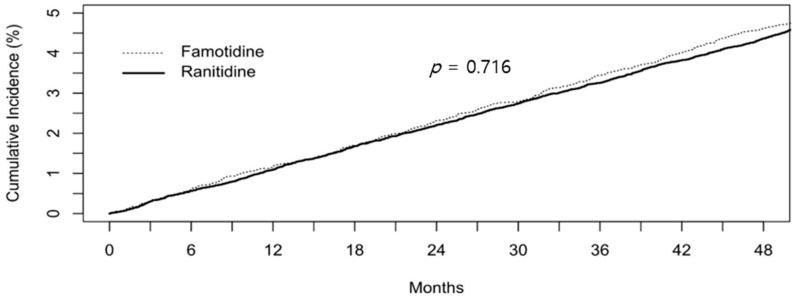
Cumulative incidence of cancer between ranitidine users and famotidine users.

When analyzed according to the cumulative duration of ranitidine and famotidine intake, there was again no difference in the overall cancer risk (*p* = 0.716, Figure 2). All 11 single cancers showed the same pattern for cumulative incidence.

## 4. Discussion

The stomach secretes gastric acid, which plays a physiological role, such as that of removing potentially harmful bacteria and aiding in digestion. However, some diseases, such as GERD and PUD, are aggravated by gastric acid, and their treatment evolved significantly with the development of H2RA in the 1970s [18]. H_2_-receptors play an important role in gastric acid secretion, and their antagonists, the H2Ras, compete with these histamines to bind H_2_-receptors and inhibit gastric acid secretion. Ranitidine, introduced in 1981, has been sold in more than 120 countries, formulated for the treatment of 222 million patients worldwide. In other words, ranitidine is one of the most extensively studied and used drugs. Although ranitidine has many side effects, such as bloating, fever, and hepatotoxicity, similar to many other medicines, there have been no reports of malignant disease associated with their use [19,20], and so many clinicians were not worried about the risk of cancer from ranitidine. However, owing to the recent detection of carcinogens in ranitidine, additional research is needed to clarify the risk of cancer from ranitidine use.

Researchers have studied NDMA formation in ranitidine [1,21], and chloramination of ranitidine has been suggested as a formulation mechanism of NDMA. The formation of NDMA is mainly determined by chloramine and precursor amine groups, both of which are highly dependent on pH [8]. The optimal pH for NDMA formation appears to be at 7–8. At lower pH, the reaction is limited because of the lack of non-protonated amines. A study conducted in 2016 by Zeng et al. reported the production of NDMA by nitrosation of ranitidine under stomach-relevant pH conditions in vitro [9]. However, the usual caveats of in vitro studies exist, and there is a lack of research on the formation of NDMA in vivo. Matsuda et al. reported that after ranitidine intake, the maximum concentration of NDMA in the stomach is 7.9 ng/mL, which is 6.6 times higher than reported before [7]. In another study, NDMA excreted in urine after ranitidine intake was 95.6 ng/mL, which was a 430-fold increase than before ranitidine use [9]. Actual systemic NDMA exposure is likely much higher than that eliminated in urine [22]. 

NDMA is a semi-volatile organic chemical that is produced by both industrial and natural processes. It is a member of N-nitrosamines, a family of potent carcinogens [23]. NDMA is not currently produced in pure form or commercially used, except for research purposes. It was formerly used to produce liquid rocket fuel, as an antioxidant, and as an additive for lubricants [24]. The most significant toxicity of NDMA is carcinogenicity, which has been studied for more than 50 years. Although sufficient epidemiological studies have not yet been reported to provide evidence for the association between NDMA and cancer development, animal studies have shown that the risk of cancer may increase with exposure to NDMA. Tumors were found to develop in the lungs, liver, kidneys, and bile ducts in animals (such as rats, mice, hamsters, and rabbits) through inhalation or oral administration [4]. It is debatable whether NDMA directly causes cancer or merely increases the predisposition or susceptibility of an individual to cancer. Genetic toxicity associated with carcinogenicity includes excessive DNA methylation, DNA fragmentation, chromosomal abnormalities, and mutation, and can also cause sperm malformations, as observed in several animal studies [25,26]. Evidence of carcinogenicity in rodents was found when administered at a dose of 10 μg/kg/day [15]. According to an FDA announcement, NDMA levels of up to 0.86 μg on the intake of 300 mg ranitidine tablets and up to 0.36 μg on the intake of 150 mg tablets were measured. The daily exposure for a 70 kg adult is estimated at approximately 0.012 μg/kg/day. Although it is impossible to apply the results of animal experiments directly to humans, the daily exposure of NDMA from ranitidine in humans is much lower than the lowest NDMA dose leading to cancer in rodents.

The primary strength of this study was the population size selected from a high quality nationwide and population-based database. There are few epidemiological studies evaluating cancer risk from ranitidine use in a large population follow-up cohort [27]. The selection of famotidine users as controls is suitable because famotidine, whose function is similar to ranitidine, lacks NDMA. Famotidine is comparatively more potent than ranitidine at gastric acid suppression. However, proton pump inhibitors are much more potent than H2RA and are widely used for gastric acid suppression [28]. Therefore, the clinical indications of ranitidine and famotidine are almost identical [5], and as all co-variates cannot be adjusted, a control group using a similar indication drug is more appropriate than the general population. In a recent Japanese study, the authors compared ranitidine/nizatidine users with other H2 blocker users. They reported that ranitidine and nizatidine did not increase the risk of cancer [27].

This study has several limitations. First, some cancer incidences and drug exposures are not included, due to study design limitations. For example, if a patient used ranitidine from January 2009 to December 2009 and was diagnosed with cancer in 2011, he would have been excluded from the study. These limitations apply equally to both groups. Since the two groups were compared under the same conditions, the results can be adopted. Second, cancer incidence was not compared with the general population. We compared SIR (standardized incidence ratio, adjusted by age and sex) for cancer in the general population and medication group. The overall SIR for all cancers was 1.22 (Ranitidine vs. famotidine 1.23 vs. 1.31 *p* < 0.001). Because of the protopathic bias, cancer incidence is higher than that of the general population. Third, the follow-up period is restricted to seven years; thus, the overall follow-up period is not long enough to assess the onset of cancer. Fourth was the lack of information about potential confounders of cancer, such as smoking and underlying diseases other than DM. Fifth, the actual exposure of NDMA was not measured and is difficult to estimate, thus actual exposure in an individual patient may be different. Sixth, medication compliance was not known and thus could be a confounding factor.

Despite these limitations, this study will assist with clarifying the suspected risk of cancer following ranitidine prescription. 

To conclude, we found no association between probable NDMA exposure through ranitidine and the short-term risk of cancer. However, further research is needed to assess the long-term cancer risk.

## Figures and Tables

**Table 1:** Baseline characteristics of the cohort of ranitidine and famotidine users.

Characteristics	Ranitidine (*n* = 88,416)	Famotidine (*n* = 10,129)
Male (n, %)	43,217 (48.9)	5307 (52.4)
Age (years, mean ± SD)	61.0 ± 11.3	59.8 ± 11.8
30–49 (years, n, %)	14,305 (16.2)	1966 (19.4)
50–59 (years, n, %)	23,356 (26.4)	2810 (27.7)
60–69 (years, n, %)	26,914 (30.4)	2850 (28.2)
70–79 (years, n, %)	23,841 (27.0)	2503 (24.7)
Diabetes mellitus	32,051 (36.3)	3501 (34.6)
Diabetes mellitus period		
≤3 years	9529 (10.8)	1006 (9.9)
≥4 years	22,522 (25.5)	2495 (24.6)
Dosage		
mg, (mean ± SD)	204,919 ± 104,366	29,579 ± 15,544
DDD (mean ± SD)	683.1 ± 347.9	739.5 ± 388.6
Cumulative duration		
12–17 months	40,557 (45.9)	4023 (39.7)
18–23 months	19,361 (21.9)	2133 (21.0)
24–29 months	11,126 (12.6)	1364 (13.5)
≥30 months	17,372 (19.6)	2609 (25.8)

DDD, Defined daily dose.

**Table 2:** Baseline characteristics of a 4:1 matched cohort (age, sex, DM, and cumulative duration) of ranitidine and famotidine users.

Characteristics	Ranitidine (*n* = 40,488)	Famotidine (*n* = 10,122)	*p*-Value
Male (n, %)	21,208 (52.4)	5302 (52.4)	>0.999
Age (years, mean ± SD)	59.9 ± 11.8	59.8 ± 11.8	0.765
30–49 (years, n, %)	7836 (19.3)	1959 (19.3)	
50–59 (years, n, %)	11,240 (27.8)	2810 (27.8)	
60–69 (years, n, %)	11,400 (28.2)	2850 (28.2)	
70–79 (years, n, %)	10,012 (24.7)	2503 (24.7)	
Diabetes mellitus	13,976 (34.5)	3494 (34.5)	>0.999
Diabetes mellitus period			>0.999
≤3 years	4020 (9.9)	1005 (9.9)	
≥4 years	9956 (24.6)	2489 (24.6)	
Dosage			
mg, mean ± SD)	220,395 ± 112,850	29578 ± 15549	
DDD (mean ± SD)	734.6 ± 376.2	739.4 ± 388.7	0.254
Cumulative duration			>0.999
12–17 months	16,092 (39.8)	4023 (39.8)	
18–23 months	8524 (21.0)	2131 (21.0)	
24–29 months	5440 (13.4)	1360 (13.4)	
≥30 months	10,432 (25.8)	2608 (25.8)	

DDD, Defined daily dose.

**Table 3:** Risk of cancer between ranitidine users and famotidine users.

Cancer	Ranitidine (*n,* %)	Famotidine (*n*, %)	*p*-Value	Hazard Ratio (95% CI)	*p*-Value
Overall	3018 (7.45)	765 (7.56)	0.739	0.99 (0.91–1.07)	0.716
Liver	400 (0.99)	117 (1.16)	0.148	0.85 (0.69–1.05)	0.133
Colorectal	359 (0.89)	92 (0.91)	0.878	0.98 (0.78–1.23)	0.832
Biliary	93 (0.23)	19 (0.19)	0.493	1.22 (0.75–2.00)	0.422
Stomach	450 (1.11)	106 (1.05)	0.616	1.06 (0.86–1.31)	0.580
Lung	473 (1.17)	119 (1.18)	0.992	0.99 (0.81–1.21)	0.951
Prostate	173 (0.43)	43 (0.42)	>0.999	1.01 (0.72–1.40)	0.974
Kidney	72 (0.18)	23 (0.23)	0.369	0.78 (0.49–1.25)	0.306
Bladder	118 (0.29)	21 (0.21)	0.181	1.41 (0.88–2.24)	0.151
Uterine	46 (0.11)	10 (0.10)	0.815	1.15 (0.58–2.28)	0.689
Breast	108 (0.27)	33 (0.33)	0.365	0.82 (0.55–1.21)	0.313
Thyroid	159 (0.39)	37 (0.37)	0.761	1.07 (0.75–1.54)	0.694

## Data Availability

The data presented in this study are available on request from the corresponding author. The data are not publicly available due to ethical restrictions.

## References

[1] Roux J.L., Gallard H., Croué J.-P., Papot S., Deborde M. (2012). Ndma formation by chloramination of ranitidine: Kinetics and mechanism. Environ. Sci. Technol..

[2] Gerecke A.C., Sedlak D.L. (2003). Precursors of N-nitrosodimethylamine in natural waters. Environ. Sci. Technol..

[3] Atsdr U. (1997). Agency for Toxic Substances and Disease Registry; Case Studies in Environmental Medicine. http://www.atsdr.cdc.gov/HEC/CSEM/csem.html.

[4] International Agency for Research on Cancer (1987). Overall Evaluations of Carcinogenicity: An Updating of IARC Monographs Volumes 1 to 42.

[5] Lipsy R.J., Fennerty B., Fagan T.C. (1990). Clinical review of histamine2 receptor antagonists. Arch. Intern. Med..

[6] El-Shaheny R., Radwan M., Yamada K., El-Maghrabey M. (2019). Estimation of nizatidine gastric nitrosatability and product toxicity via an integrated approach combining hilic, in silico toxicology, and molecular docking. J. Food Drug Anal..

[7] Matsuda J., Hinuma K., Tanida N., Tamura K., Ohno T., Kano M., Shimoyama T. (1990). N-nitrosamines in gastric juice of patients with gastric ulcer before and during treatment with histamine H2-receptor antagonists. Gastroenterol. Jpn..

[8] Shen R., Andrews S.A. (2013). Formation of NDMA from ranitidine and sumatriptan: The role of pH. Water Res..

[9] Zeng T., Mitch W.A. (2016). Oral intake of ranitidine increases urinary excretion of N-nitrosodimethylamine. Carcinogenesis.

[10] Michaud D.S., Mysliwiec P.A., Aldoori W., Willett W.C., Giovannucci E. (2004). Peptic ulcer disease and the risk of bladder cancer in a prospective study of male health professionals. Cancer Epidemiol. Prev. Biomark..

[11] Vermeer I.T., Engels L.G., Pachen D.M., Dallinga J.W., Kleinjans J.C., Van Maanen J.M. (2001). Intragastric volatile N-nitrosamines, nitrite, pH, and Helicobacter pylori during long-term treatment with omeprazole. Gastroenterology.

[12] Pottegård A., Kristensen K.B., Ernst M.T., Johansen N.B., Quartarolo P., Hallas J. (2018). Use of n-nitrosodimethylamine (ndma) contaminated valsartan products and risk of cancer: Danish nationwide cohort study. BMJ.

[13] Song S.O., Jung C.H., Song Y.D., Park C.-Y., Kwon H.-S., Cha B.S., Park J.-Y., Lee K.-U., Ko K.S., Lee B.-W. (2014). Background and data configuration process of a nationwide population-based study using the korean national health insurance system. Diabetes Metab. J..

[14] Lijinsky W., Reuber M.D. (1984). Carcinogenesis in rats by nitrosodimethylamine and other nitrosomethylalkylamines at low doses. Cancer Lett..

[15] Peto R., Gray R., Brantom P., Grasso P. (1984). Nitrosamine carcinogenesis in 5120 rodents: Chronic administration of sixteen different concentrations of ndea, ndma, npyr and npip in the water of 4440 inbred rats, with parallel studies on ndea alone of the effect of age of starting (3, 6 or 20 weeks) and of species (rats, mice or hamsters). IARC Sci. Publ..

[16] Seong S.C. (2015). National Health Insurance System of Korea. http://www.google.com/url?sa=t&rct=j&q=&esrc=s&source=web&cd=&ved=2ahUKEwiyneiVi4DuAhVHPnAKHRqGAnAQFjAAegQIAxAC&url=http%3A%2F%2Fwww.kobia.kr%2Fskin%2Fbbs%2Fdownloads_e2%2Fdownload.php%3Ftbl%3Dpolicy_report%26no%3D401&usg=AOvVaw2UvT38upoP4Ka4J_a34H3J.

[17] WHO Collaborating Centre for Drug Statistics Methodology (2013). Guidelines for ATC Classification and DDD Assignment. www.whocc.no/filearchive/publications/1_2013guidelines.pdf.

[18] Black J., Duncan W., Durant C.J., Ganellin C.R., Parsons E. (1972). Definition and antagonism of histamine h2-receptors. Nature.

[19] Mills J.G., Koch K.M., Webster C., Sirgo M.A., Fitzgerald K., Wood J.R. (1997). The safety of ranitidine in over a decade of use. Aliment. Pharmacol. Ther..

[20] Wormsley K.G. (1993). Safety profile of ranitidine: A review. Drugs.

[21] Liu Y.D., Selbes M., Zeng C., Zhong R., Karanfil T. (2014). Formation mechanism of ndma from ranitidine, trimethylamine, and other tertiary amines during chloramination: A computational study. Environ. Sci. Technol..

[22] Spiegelhalder B., Eisenbrand G., Preussmann R. (1982). Urinary excretion of n-nitrosamines in rats and humans. IARC Sci. Publ..

[23] Fan C.-C., Lin T.-F. (2018). N-nitrosamines in drinking water and beer: Detection and risk assessment. Chemosphere.

[24] Mulhern R.E. (2016). Removal of N-Nitrosodimethylamine and Otherdisinfection by-Product Precursors from Tertiary Wastewater Effluent by Activatedcarbon. Ph.D. Thesis.

[25] Schwarzenegger A., Adams L.S., Denton J.E. (2006). N-nitrosodimethylamine. https://www.josorge.com/publications/Citations/Toxicology/021.pdf.

[26] Bartsch H., O’Neill I.K. (1988). Ninth International Meeting on N-Nitroso Compounds: Exposures, Mechanisms, and Relevance to Human Cancer.

[27] Iwagami M., Kumazawa R., Miyamoto Y., Ito Y., Ishimaru M., Morita K., Hamada S., Tamiya N., Yasunaga H. (2020). Risk of cancer in association with ranitidine and nizatidine vs other h2 blockers: Analysis of the japan medical data center claims database 2005–2018. Drug Saf..

[28] Ducrotté P., Guillemot F., Elouaer-Blanc L., Hirschauer C., Thorel J.M., Petit A., Hochain P., Michel P., Cortot A., Colin R. (1994). Comparison of omeprazole and famotidine on esophageal ph in patients with moderate to severe esophagitis: A cross-over study. Am. J. Gastroenterol..

